# The deubiquitinase CYLD inhibits thrombin-induced p38 MAPK–p65 NF-κB signaling and inflammatory cytokine production to suppress triple-negative breast cancer progression

**DOI:** 10.1016/j.jbc.2026.113341

**Published:** 2026-07-16

**Authors:** Julio M. Pimentel, Oye Bosompra, JoAnn Trejo

**Affiliations:** 1 Department of Pharmacology, School of Medicine, University of California, San Diego, La Jolla, California, USA; 2 Biomedical Sciences Graduate Program, University of California, San Diego, La Jolla, California, USA; 3 Moores Cancer Center, University of California San Diego, La Jolla, California, USA

**Keywords:** GPCR, IL-6, IL-8, inflammation, interleukin, PAR1, protease-activated receptor-1

## Abstract

Triple-negative breast cancer (TNBC) is an aggressive subtype characterized by chronic inflammation with limited therapeutic options. Although thrombin activation of protease-activated receptor-1 (PAR1) promotes ubiquitin-dependent signaling and inflammatory responses in endothelial cells, its contribution to inflammatory effects in TNBC remain poorly understood. Here, we show that loss of the deubiquitinase cylindromatosis (CYLD) markedly increases basal phosphorylation of p38 mitogen-activated protein kinase and p65 nuclear factor kappa B subunit to levels comparable to thrombin stimulation, without further enhancement by thrombin, indicating that CYLD primarily restrains constitutive inflammatory activity. We also show that thrombin-activation of PAR1 promotes p38 signaling through an autophosphorylation-dependent mechanism in TNBC, leading to p65 nuclear factor kappa B subunit phosphorylation and nuclear accumulation independent of canonical inhibitor of κBα degradation. This non-canonical signaling drives expression of pro-inflammatory interleukin (IL) cytokines including IL-6, IL-8, IL-1α, and IL-1β. Functionally, IL-6 and IL-8 contribute to thrombin-induced TNBC proliferation and migration, respectively. Together, these findings define a novel CYLD-regulated PAR1–p38–p65 signaling axis that promotes inflammatory cytokine production and tumor-associated phenotypes such as cell proliferation and migration, identifying multiple druggable nodes for therapeutic targeting in aggressive breast cancers.

Breast cancer is the most commonly diagnosed cancer and second-leading cause of death among women in the United States (1). Among the major molecular subtypes, triple-negative breast cancer (TNBC) is defined by the absence of the estrogen receptor, progesterone receptor, and human epidermal growth factor receptor 2 expression and is associated with aggressive behavior, early recurrence, and poor clinical outcomes (2, 3). Chemotherapy remains the standard of care for TNBC, but resistance often develops, driving metastatic progression and limiting durable disease control (4, 5). Therefore, defining the molecular mechanisms driving TNBC progression is essential for identifying new targets and developing more effective therapies.

Chronic inflammation drives TNBC progression and metastasis (6, 7). Elevated cytokines, including interleukin-6 (IL-6) and IL-8, promote survival, migration, and drug resistance and are associated with poor clinical outcomes (6, 7). These responses are mediated in part by stress-responsive pathways such as p38 mitogen-activated protein kinase (MAPK), which promote aggressive phenotypes (8, 9, 10, 11, 12). G protein-coupled receptors (GPCRs) are key upstream regulators of inflammatory signaling (11, 13, 14, 15, 16). However, the mechanisms linking GPCR signaling to p38 activation and inflammatory cytokine production in TNBC remain poorly defined.

Protease-activated receptor-1 (PAR1) is a thrombin-activated GPCR that is highly expressed in TNBC and promotes pro-survival signaling and metastasis (17, 18, 19). In endothelial and HeLa cells, thrombin induces non-canonical ubiquitin-dependent transforming growth factor beta-activated kinase binding protein-1 (TAB1) and TAB2 recruitment that leads to p38 MAPK autophosphorylation, activation and induction of IL-6 expression (13, 20). Although canonical thrombin-activated PAR1-G protein signaling initiates this pathway from the plasma membrane, ubiquitinated PAR1 in complex with TAB1/TAB2 sustains inflammatory signaling at the endosome (13, 20, 21). However, whether thrombin-activation of PAR1 engages p38 pro-inflammatory signaling in TNBC and its functional impact remain unclear.

Ubiquitination is a dynamic post-translational modification controlled by deubiquitinases (DUBs) that shape signaling outputs by modifying ubiquitin chain topology (14). Using an siRNA library screen targeting 96 DUBs, we previously identified the K63-specific deubiquitinase CYLD as a key negative regulator of PAR1-mediated p38 MAPK activation in endothelial and HeLa cells (21), indicating that ubiquitin editing constrains non-canonical PAR1 inflammatory signaling. CYLD functions as a deubiquitinase has a prominent role in the regulation of nuclear factor kappa B (NF-κB) signaling (22). However, the role of CYLD in TNBC, including regulation of activated PAR1-p38 and -NF-κB signaling and the impact on tumor progression remains unclear.

Here, we identify CYLD as a critical negative regulator of thrombin-activated PAR1-p38-p65 NF-κB subunit signaling axis in TNBC cells. We demonstrate that thrombin activates p38 *via* autophosphorylation, driving p65 activation and nuclear translocation independently of inhibitor of κBα (IκBα) degradation. Furthermore, CYLD loss amplifies p38 and p65 phosphorylation, leading to sustained inflammatory cytokine production and enhanced TNBC proliferation and migration. Collectively, these findings reveal a previously unrecognized form of a non-canonical GPCR signaling axis that drives inflammatory cancer progression and identifies multiple therapeutically actionable nodes in aggressive TNBC.

## Results

### Thrombin induces PAR1-dependent p38 MAPK autophosphorylation in TNBC cells

**Figure 1:**
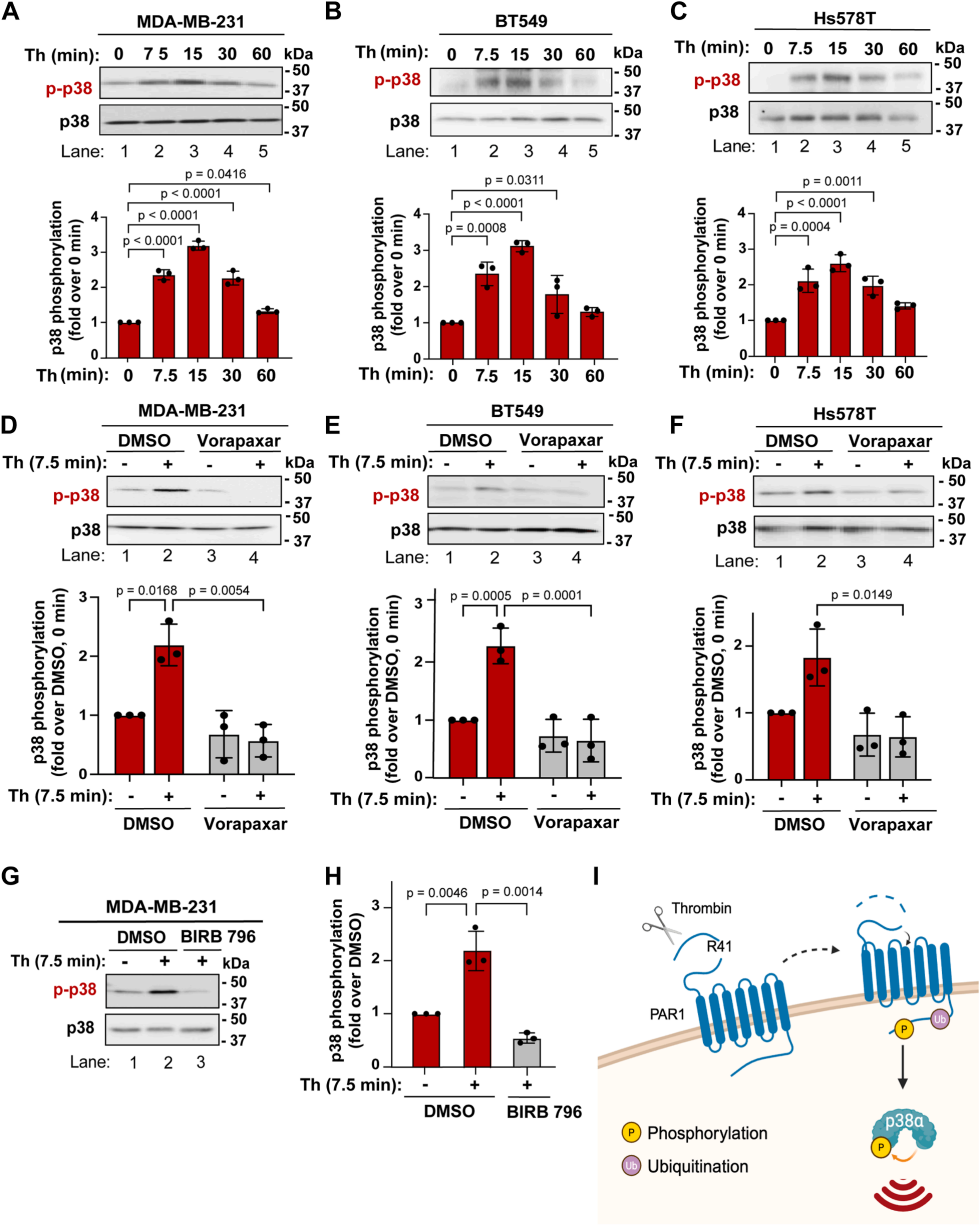
**Thrombin/PAR1 activates p38 MAPK signaling *via* autophosphorylation in TNBC cells.***A–C,* TNBC MDA-MB-231, BT549, and Hs578T cell lines were stimulated with 10 nM thrombin (Th) for the indicated times, lysed and immunoblotted as indicated. Changes in phospho-p38 were quantified after normalization to total p38. Data (mean ± S.D., n = 3 independent biological replicates) are expressed as fold over 0 min and analyzed by one-way ANOVA. *D*–*F,* cells were incubated with the PAR1 antagonist vorapaxar (10 μM) or DMSO vehicle control for 1 h prior to stimulation with Th for 7.5 min. Cell lysates were processed as described above. Data (mean ± S.D., n = 3 independent biological replicates) are expressed as fold over DMSO and analyzed by two-way ANOVA. *G and H,* cells were pretreated with either p38 inhibitor BIRB 796 (50 nM) or DMSO vehicle control for 1 h, stimulated with Th and changes in phospho-p38 quantified after normalization as described above. Data (mean ± S.D., n = 3 independent biological replicates) are expressed as fold change over DMSO and analyzed by two-way ANOVA. *I,* schematic model of Th-activated PAR1-mediated p38 autophosphorylation and activation in TNBC cells. TNBC, triple-negative breast cancer; Th, thrombin.

Thrombin has previously been shown to activate PAR1-p38 MAPK inflammatory signaling in endothelial cells (13, 21). To determine whether thrombin induces inflammatory signaling in TNBC, we examined phosphorylation of p38 in human invasive ductal breast carcinoma cells, which exhibit high PAR1 expression (18, 23). Across three TNBC cell lines, thrombin induced a robust increase in p38 Thr180/Tyr182 phosphorylation that peaked between 7.5 and 15 min (Fig. 1, *A*–*C*, lane 2–3 *versus* 1), consistent with previous reports using other cell types (13, 20, 21). To determine whether this response is mediated by PAR1, we used the PAR1-selective antagonist vorapaxar, which markedly attenuated thrombin-induced p38 phosphorylation across all cell lines compared to vehicle control (Fig. 1, *D*–*F*, lanes 3–4 *versus* 1–2), identifying PAR1 as the primary effector of thrombin signaling. Thrombin-mediated PAR1 activation of p38 occurs through autophosphorylation in other cell types (13, 24), thus we next examined whether this mechanism exists in TNBC cells. MDA-MB-231 cells were pretreated with the p38 inhibitor BIRB 796, which blocks p38 activity and autophosphorylation but not upstream MAPK kinase-mediated p38 phosphorylation. Thrombin-induced p38 phosphorylation was virtually abolished in cells pretreated with BIRB 796 (Fig. 1*G*, lane 2 *versus* 3; Fig. 1*H*), indicating that autophosphorylation is required for p38 phosphorylation. These results suggest that thrombin induces activation of p38 in TNBC cells through a PAR1-dependent, autophosphorylation mechanism (Fig. 1*I*).

### Thrombin-induced p38 phosphorylation drives p65 phosphorylation and nuclear translocation

**Figure 2:**
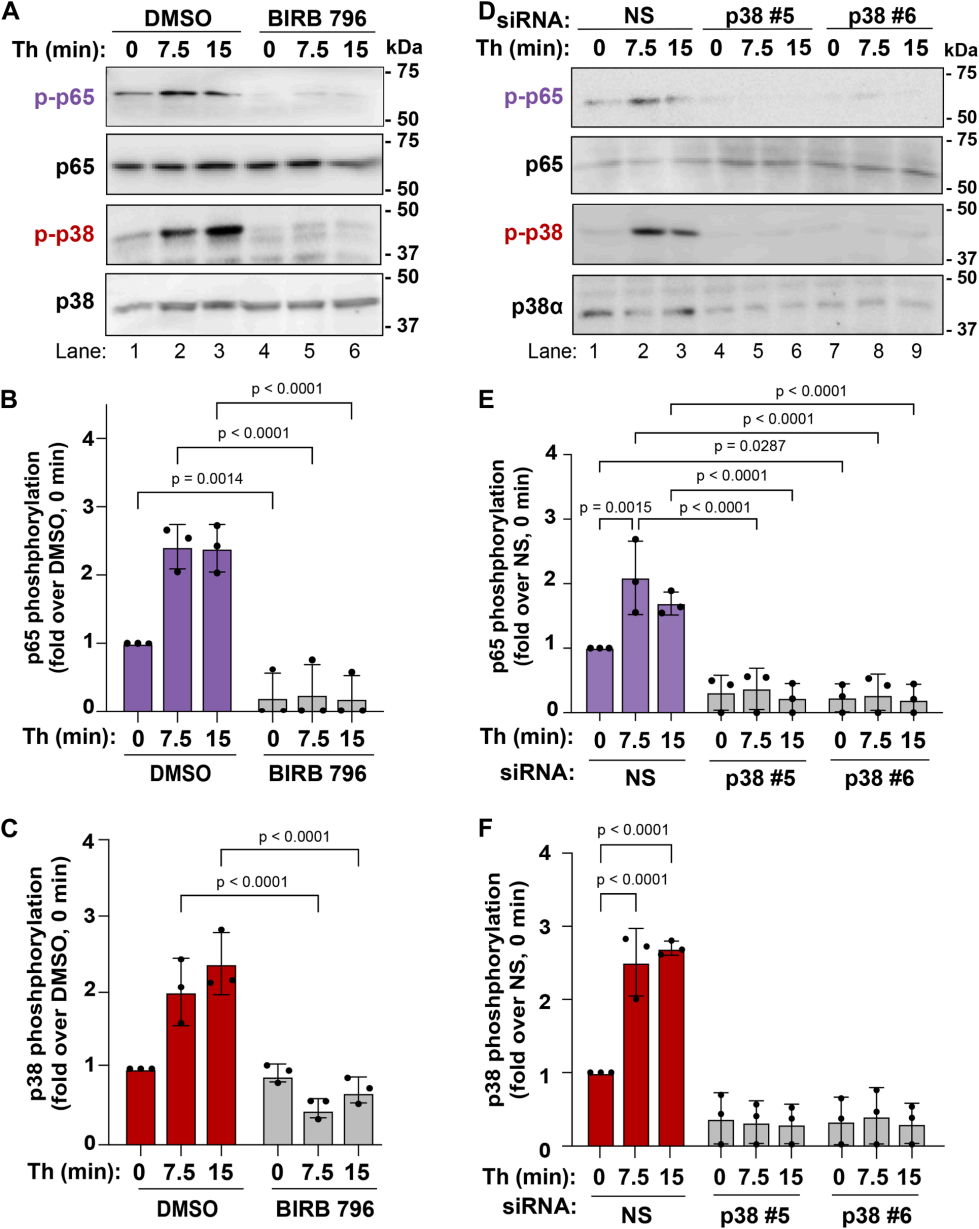
**Thrombin-induced phosphorylation of the p65 NF-κB subunit is dependent on p38 activation.***A,* MDA-MB-231 cells incubated with BIRB 796 (50 nM) or DMSO vehicle control were stimulated with 10 nM thrombin (Th) for various times and immunoblotted as indicated. *B and C,* changes in phospho-p38 and phospho-p65 were quantified after normalization to total p38 or p65, respectively. Data (mean ± S.D., n = 3 independent biological replicates) are expressed as fold change over control at 0 min and analyzed by two-way ANOVA. *D,* cells were transfected separately with two different p38-specific siRNAs (#5 or #6) or NS siRNA, stimulated with Th and immunoblotted as indicated. *E and F,* phospho-p38 and phospho-65 were normalized, quantified and data (mean ± S.D., n = 3 independent biological replicates) expressed as fold over NS control at 0 min and analyzed by two-way ANOVA. Th, thrombin; NS, non-specific.

The p65 NF-κB subunit is a central transcriptional activator of pro-inflammatory gene expression (25), and can be directly regulated by p38 (25, 26, 27). However, it remains unclear whether thrombin-induced p38 phosphorylation drives p65 phosphorylation or *vice versa*. To determine whether p38 regulates p65 phosphorylation, MDA-MB-231 cells were pretreated with the p38 inhibitor BIRB 796 prior to thrombin stimulation. Inhibition of p38 significantly reduced thrombin-induced p65 Ser536 phosphorylation (Fig. 2*A*, lanes 4–6 *versus* 1–3; Fig. 2, *B* and *C*), supporting a role for p38 acting upstream of p65 phosphorylation. To validate this finding, we used an siRNA approach to knockdown p38 expression in MDA-MB-231 cells. Depletion of p38 with two different siRNAs in MDA-MB-231 cells significantly reduced thrombin-stimulated p65 phosphorylation compared to non-specific siRNA control (Fig. 2*D*, lanes 4–9 *versus* 1–3; Fig. 2, *E* and *F*). These results support a role for p38 acting upstream of p65 phosphorylation in response to thrombin.

**Figure 3:**
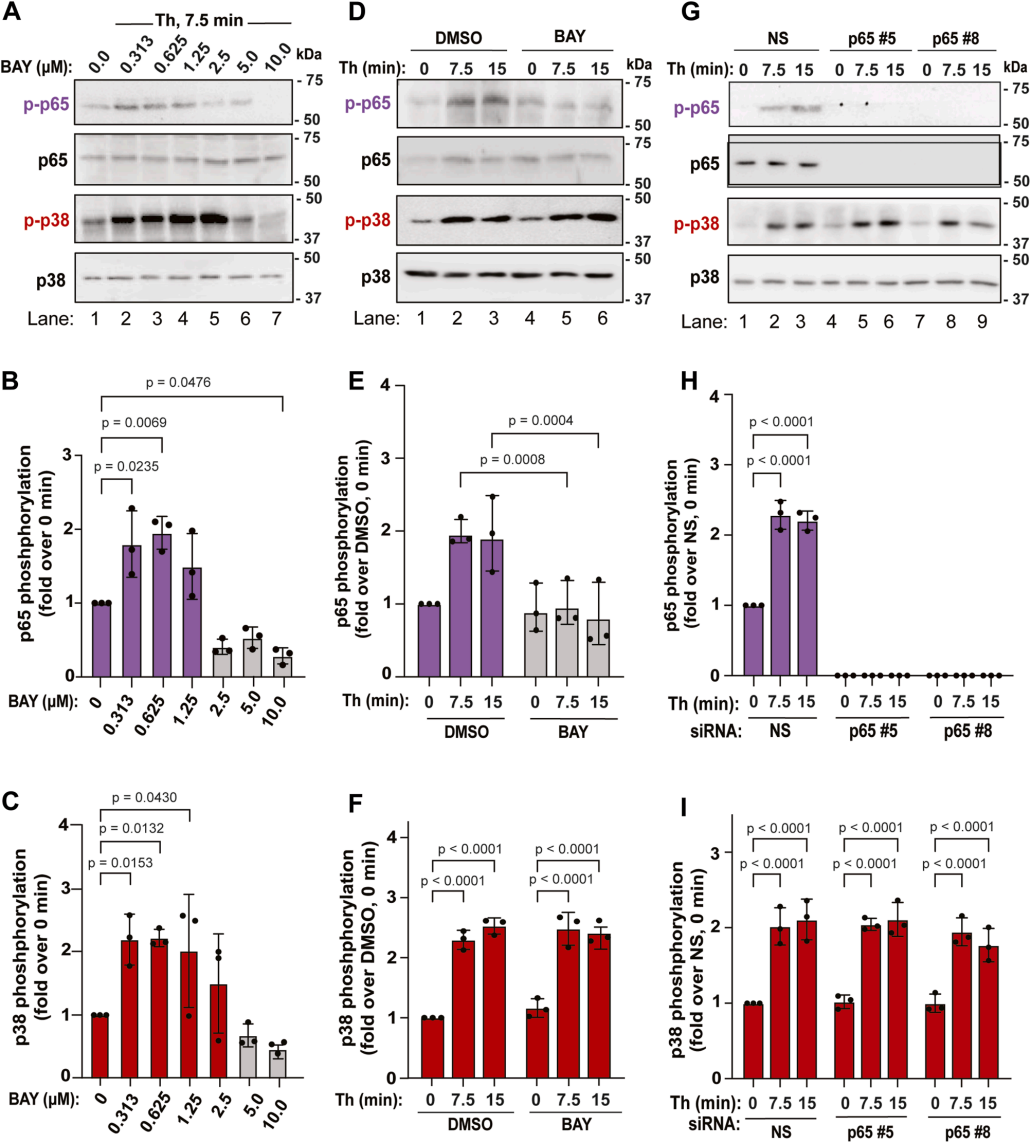
**Thrombin-induced p38 activation occurs independently of p65.***A,* MDA-MB-231 cells were pretreated with various concentrations of the p65 inhibitor BAY 11-7082 for 1 h, stimulated with 10 nM thrombin (Th) for 7.5 min and immunoblotted as indicated. *B and C,* changes in phospho-p65 and phospho-p38 were normalized to total p65 or p38, respectively, quantified and data (mean ± S.D., n = 3 independent biological replicates) expressed as fold over 0 min control and analyzed by two-way ANOVA. *D,* cells were pretreated with BAY (2.5 μM) or vehicle for 1 h, stimulated with Th stimulated for 7.5 or 15 min. *E and F,* data (mean ± S.D., n = 3 independent biological replicates) were expressed as fold over DMSO, 0 min and analyzed by two-way ANOVA. *G,* cells were transfected separately with two different p65-specific siRNAs (#5 and #8) or NS siRNA, stimulated with Th and immunoblotted as indicated. *H and I,* data (mean ± S.D., n = 3 independent biological replicates) were expressed as fold over NS, 0 min and analyzed by two-way ANOVA as described above. Th, thrombin; NS, non-specific.

To determine whether p65 NF-κB subunit regulates p38 phosphorylation, MDA-MB-231 cells were pretreated with increasing concentrations of the p65 inhibitor BAY 11-7082 prior to thrombin stimulation. At the 2.5 μM concentration, BAY effectively suppressed thrombin-induced p65 phosphorylation without affecting p38 phosphorylation (Fig. 3*A*, lane 5 *versus* 2–4; Fig. 3, *B* and *C*), indicating that p65 signaling is not required for p38 activation. At higher concentrations, BAY reduced p38 phosphorylation (Fig. 3*A*, lanes 6–7 *versus* 2–5; Fig. 3, *B* and *C*), suggesting off-target effects. Thus, 2.5 μM BAY was used for subsequent studies. Thrombin-stimulated p65 phosphorylation over time further revealed that BAY at 2.5 μM effectively blocked p65 phosphorylation without altering phosphorylation of p38 (Fig. 3*D*, lanes 4–6 *versus* 1–3: Fig. 3, *E* and *F*). Additionally, siRNA-mediated knockdown of p65 using two different siRNAs similarly abolished thrombin-induced p65 phosphorylation but did not affect p38 phosphorylation compared to non-specific siRNA controls (Fig. 3*G*, lanes 4–9 *versus* 1–3; Fig. 3, *H* and *I*). These findings indicate that p38 functions upstream of p65 phosphorylation following thrombin stimulation in MDA-MB-231 cells.

**Figure 4:**
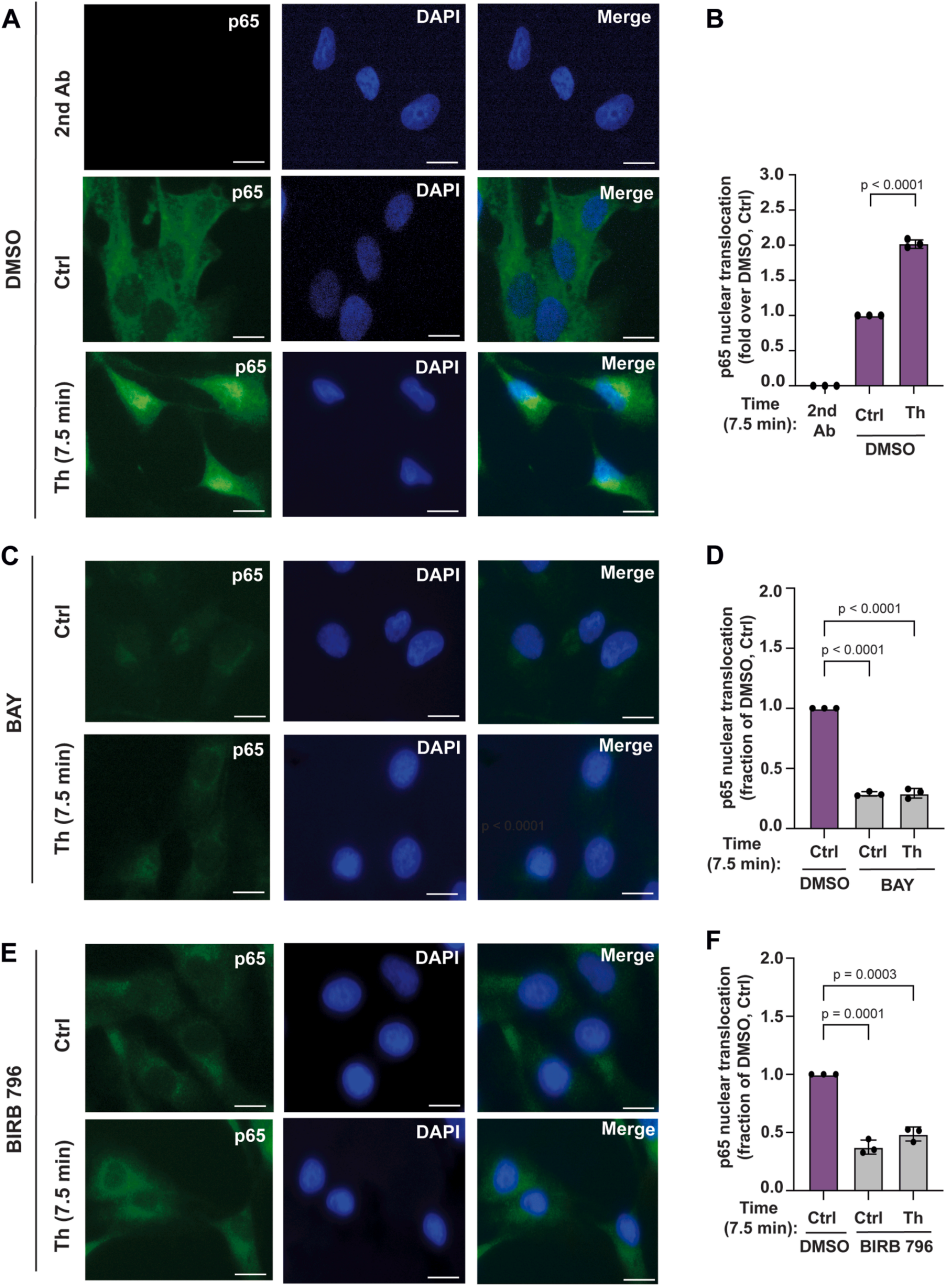
**Thrombininduces p65 activation and nuclear translocation *via* p38 autophosphorylation.** MDA-MB-231 cells were incubated with fasudil (30 μM for 1 h) and then pretreated with either *(A and B)* DMSO vehicle, *(C and D)* BAY 11-7082 (2.5 μM) or *(E and F)* BIRB 796 (50 nM) for 1 h prior to 10 nM thrombin (Th) stimulation for 7.5 min *(A, C, E)* Cells were fixed and incubated with secondary antibody (second Ab) goat-anti-rabbit Alexa-488 only or anti-p65 antibody (*green*) followed by second Ab and then stained with DAPI (*blue*) to detect nuclei and imaged by confocal microscopy. Nuclear p65 translocation was quantified from six images per condition from three independent experiments. The data (mean ± S.D., n = 3 independent biological replicates) are expressed as the fold over DMSO control *(B)* or fraction of DMSO control *(D and F)* following normalization to the Alexa Fluor 488 secondary antibody control. Statistical significance was determined by two-way ANOVA. The scale bar represents, 10 μm. Th, thrombin.

To determine whether thrombin-induced p38 phosphorylation drives downstream NF-κB activity, we assessed p65 nuclear translocation by confocal microscopy. MDA-MB-231 cells were pretreated with the Rho-associated protein kinase inhibitor fasudil to reduce cell contraction and preserve cell morphology. Under control conditions, p65 was predominantly cytoplasmic and exhibited robust nuclear translocation upon 7.5 min of thrombin stimulation (Fig. 4, *A* and *B*). Thrombin-induced p65 nuclear translocation was abolished in cells treated with BAY (Fig. 4, *C* and *D*). No signal was detected in cells immunostained with secondary antibody (Fig. 4*A*). To evaluate the role of p38, cells were pretreated with BIRB 796 and stimulated with thrombin, which significantly reduced p65 nuclear translocation (Fig. 4, *C*–*F*) compared to DMSO vehicle treated cells stimulated with thrombin (Fig. 4*A*). Thus, thrombin-induced p38 activation functions upstream of p65 and drives p65 nuclear localization in TNBC cells.

### p38 and p65 form a preexisting complex in which thrombin enhances phosphorylation independently of inhibitor of κBα degradation

**Figure 5:**
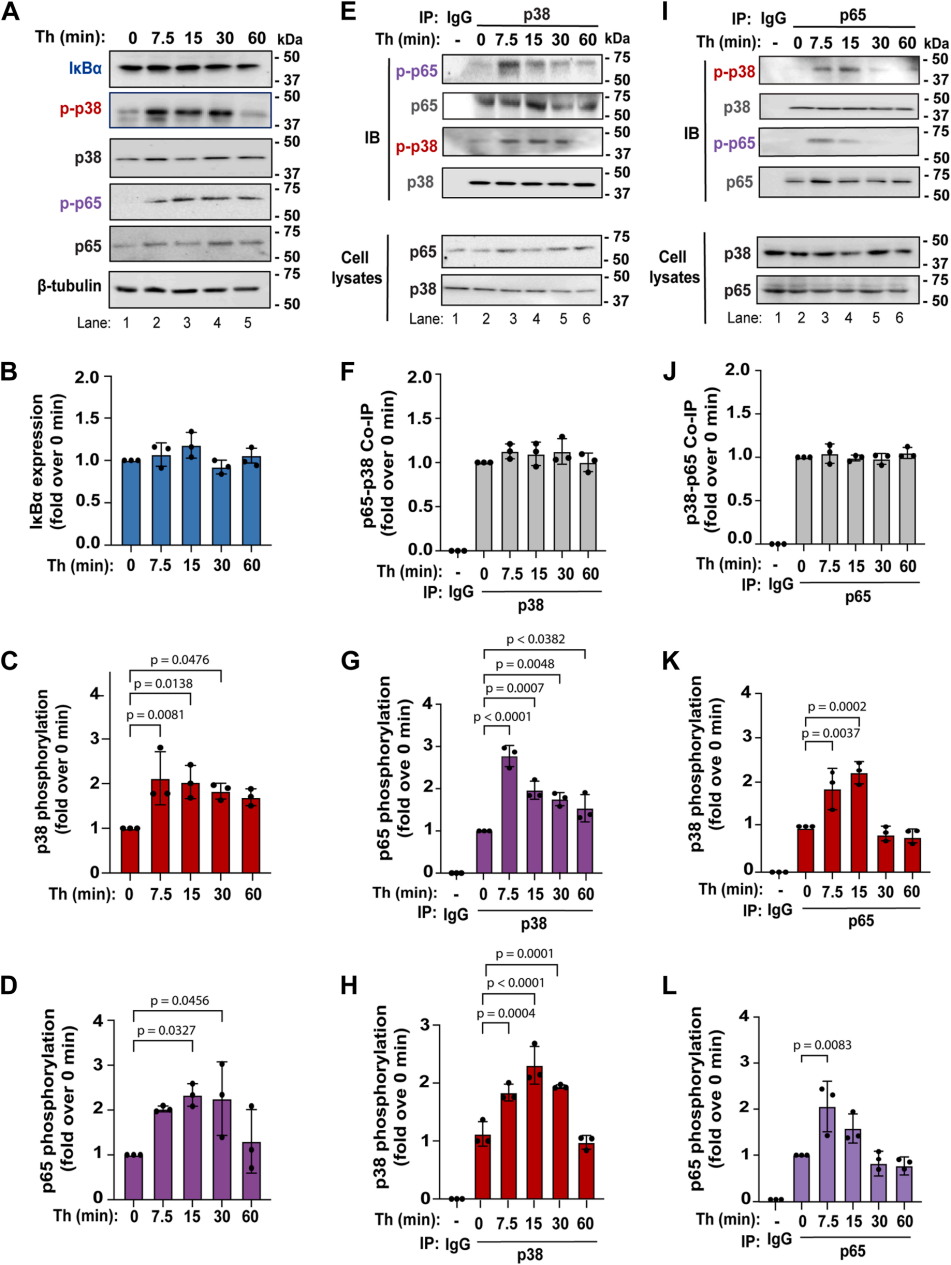
**Thrombinpromotes phosphorylation of a pre-formed p38-p65 complex.***A,* MDA-MB-231 cells were stimulated with 10 nM thrombin (Th) for various times, lysed and immunoblotted as indicated. *B,* changes in IκBα expression were determined after normalization to β-tubulin *(B)*, whereas phospho-p38 *(C)* and phospho-p65 *(D)* were normalized to their respective total protein and quantified. Data (mean ± S.D., n = 3 independent biological replicates) are presented as fold over 0 min and analyzed by one-way ANOVA. *E,* cells were stimulated with Th at various times, lysed, immunoprecipitated and immunoblotted as indicated. *F,* changes in anti-p38 antibody immunoprecipitated p65-p38 complex, *(G)* phospho-p65, and *(H)* phospho-p38 were quantified after normalization to their respective total protein and expressed as fold over 0 min. Data (mean ± S.D., n = 3 independent biological replicates) were analyzed by one-way ANOVA. *I,* cells were treated with Th and processed as described above. *J,* changes in anti-p65 antibody immunoprecipitated p38-p65 complex, *(K*) phospho-p38 and *(L)* phospho-p65 were normalized to their respective total protein and quantified. Data (mean ± S.D., n = 3 independent biological replicates) are presented as fold over 0 min and analyzed by one-way ANOVA. Th, thrombin.

The mechanism by which thrombin-induced p38 activation regulates p65 phosphorylation remains unclear. Although canonical NF-κB activation involves IκBα degradation (28), the expression of IκBα was unchanged following thrombin stimulation in MDA-MB-231 cells. Despite a lack of IκBα degradation, thrombin-induced robust p38 and p65 phosphorylation at 7.5 and 15 min (Fig. 5*A*, lanes 1–5; Fig. 5, *B*–*D*), suggesting a non-canonical activation mechanism of NF-κB. To assess whether p38 associates with p65, we performed co-immunoprecipitation (co-IP) assays using lysates from cells treated with or without thrombin. In unstimulated conditions, a p38-p65 complex was detected basally in anti-p38 antibody co-IPs but not in IgG control (Fig. 5*E*, lanes 1 *versus* 2: Fig. 5*F*). Moreover, thrombin induced robust phosphorylation of p38 and p65 proteins within the complex (Fig. 5*E*, lanes 2 *versus* 3–6; Fig. 5, *F*–*H*). Similar results were observed in reciprocal co-IPs using an anti-p65 antibody (Fig. 5*I*, lanes 1–6; Fig. 5, *J*–*L*). Together, these findings suggest that p38 and p65 form a pre-existing complex that can be activated by thrombin-induced phosphorylation independent of canonical IκBα degradation.

### CYLD restrains basal p38 autophosphorylation and activation of p65 NF-κB subunit phosphorylation in TNBC

**Figure 6:**
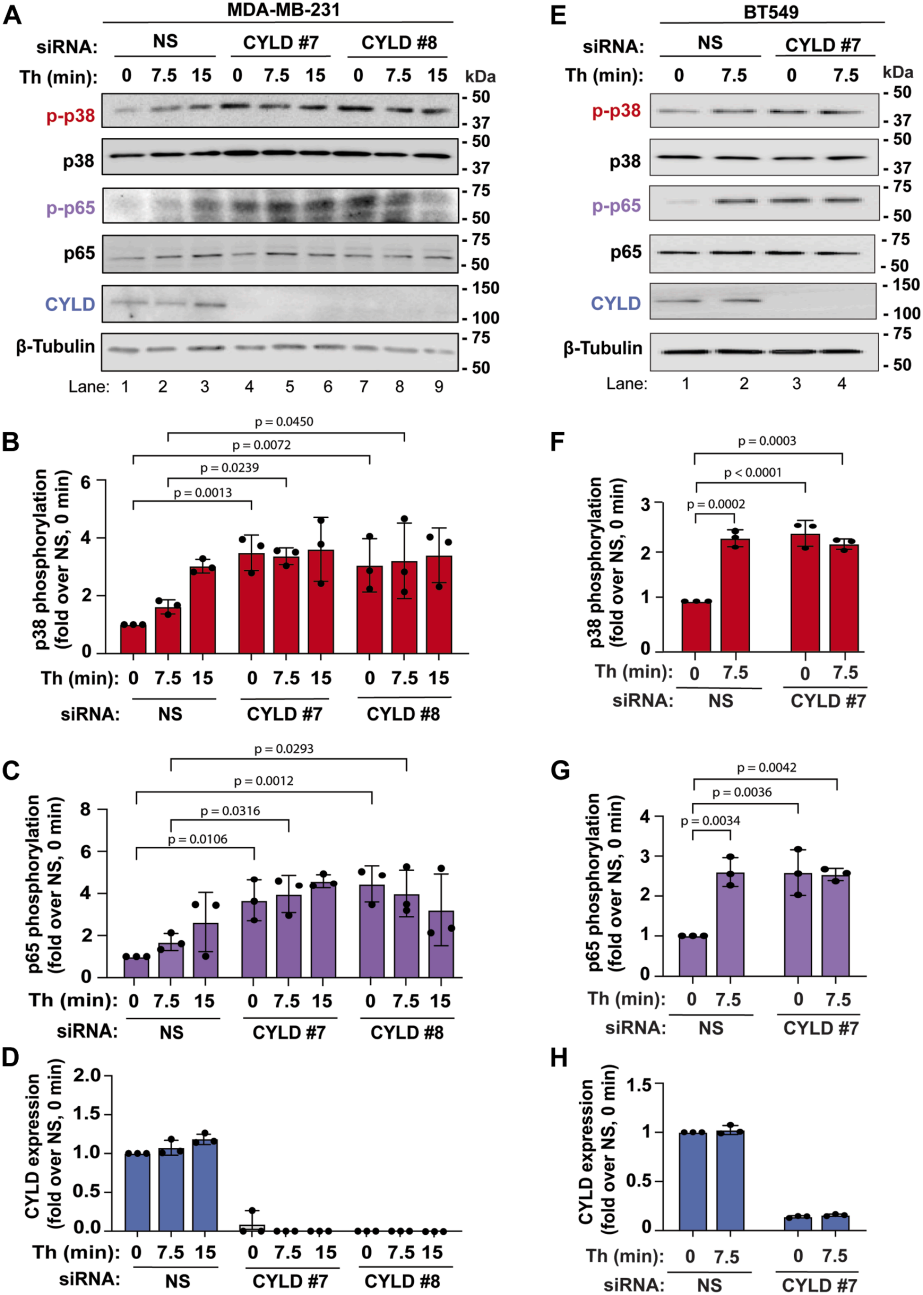
**Thrombin-induced p38 and p65 phosphorylation is enhanced in CYLD depleted cells.***A,* MDA-MB-231 cells transfected either with NS or two different CYLD siRNAs (#7 and #8) were stimulated with 10 nM thrombin (Th) for various times. Cells were lysed and immunoblotted as indicated. *B and C,* changes in phospho-p38 and phospho-p65 were normalized to their respective total protein and quantified. *D,* CYLD was normalized to β-tubulin and quantified. Data (mean ± S.D., n = 3 independent biological replicates) are expressed as fold over NS, 0 min and analyzed by two-way ANOVA. *E,* BT549 cells transfected with NS or CYLD #7 siRNA were stimulated with Th, lysed and immunoblotted as indicated. *F–H,* changes in phospho-p38, phospho-p65, and CYLD were determined as described above. Data (mean ± S.D., n = 3 independent biological replicates) are expressed as fold over NS, 0 min and analyzed by two-way ANOVA. CYLD, cylindromatosis; NS, non-specific; Th, thrombin.

The deubiquitinase CYLD is known to regulate the NF-κB signaling pathway (22, 29) and suppress thrombin-induced p38 MAPK phosphorylation in endothelial cells (21). However, it is not known if CYLD regulates thrombin-stimulated p38 and p65 signaling in TNBC. To test this, MDA-MB-231 cells were transiently transfected with two different CYLD-specific siRNAs and stimulated with thrombin. Depletion of CYLD increased basal phosphorylation of p38 and p65 detected at 0 min compared to non-specific siRNA control (Fig. 6*A*, lanes 1 *versus* 4 and 7; Fig. 6, *B*–*D*), with no further increase upon thrombin stimulation (Fig. 6*A*, lanes 4 *versus* 5–6 and lanes 7 *versus* 8–9). To determine whether the effect was observed in other TNBC cell models, we examined BT549 invasive breast carcinoma. Like in MDA-MB-231 cells, CYLD depletion significantly increased basal phosphorylation of p38 and p65 (Fig. 6*E*, lanes 1 *versus* 3; Fig. 6, *F*–*H*) and was not further enhanced by thrombin treatment (Fig. 6*E*, lanes 2 *versus* 3–4; Fig. 6, *F*–*H*), suggesting that basal phosphorylation is elevated to near-maximal levels.

**Figure 7:**
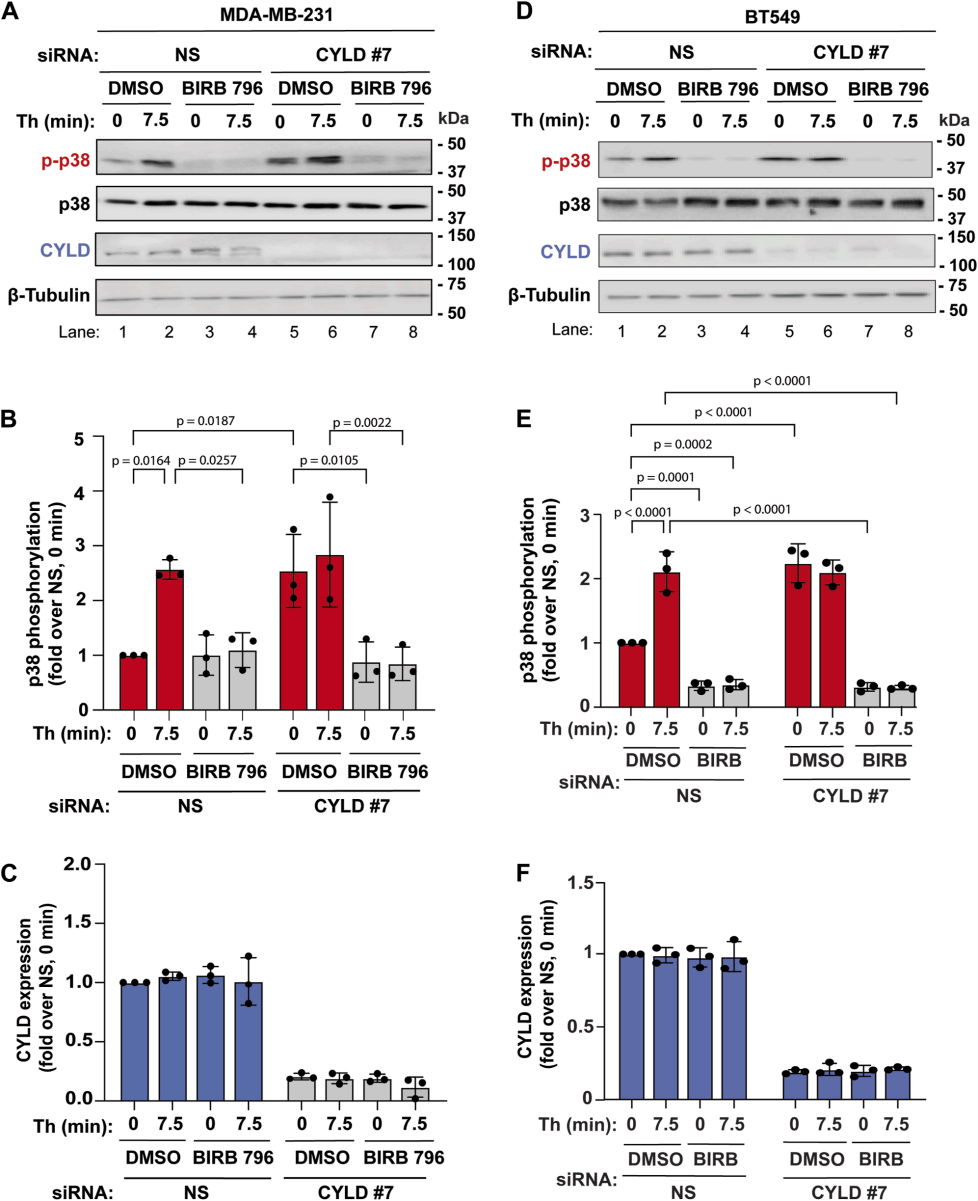
**Thrombin-induced and CYLDdepletion-induced elevated p38 phosphorylationthrough autophosphorylation.***A,* MDA-MB-231 cells transfected with NS or CYLD #7 siRNA were pretreated with 50 nM BIRB 796 or DMSO for 1 h, incubated with 10 nM thrombin (Th), lysed and immunoblotted as indicated. *B* and *C*, Changes in phospho-p38 and CYLD expression were determined after normalization to total p38 or β-tubulin, respectively. Data (mean ± S.D., n = 3 independent biological replicates) are expressed as fold over NS, at 0 min and analyzed by two-way ANOVA. *D,* BT549 cells transfected with NS or CYLD #7 siRNA treated with either BIRB 796 or DMSO were stimulated with thrombin, lysed and immunoblotted as indicated. *E and F,* changes in phospho-p38 and CYLD expression were determined as described above and data (mean ± S.D., n = 3 independent biological replicates) were analyzed by two-way ANOVA. CYLD, cylindromatosis; NS, non-specific.

To determine whether enhanced basal p38 phosphorylation depends on autophosphorylation, MDA-MB-231 cells were treated with the p38 inhibitor BIRB 796 prior to thrombin stimulation. BIRB 796 reduced both basal and thrombin-induced p38 phosphorylation in control cells (Fig. 7*A*, lanes 3–4 *versus* 1–2; Fig. 7, *B* and *C*) and suppressed elevated basal phosphorylation in CYLD-depleted cells regardless of thrombin treatment (Fig. 7*A*, lanes 7–8 *versus* 5–6; Figs. 7, *B* and *C*). Similar effects were observed in BT549 cells, where BIRB 796 blocked thrombin-induced p38 phosphorylation as well as elevated p38 phosphorylation observed in CYLD knockdown cells (Fig. 7*D*, lanes 3–4 *versus* 1–2 and 7–8 *versus* 5–6; Fig. 7, *E* and *F*). The results indicate that elevated p38 phosphorylation observed in CYLD depleted cells occurs through an autophosphorylation-dependent mechanism. Together, these results indicate that CYLD negatively regulates both basal and thrombin-stimulated p38 and p65 phosphorylation in TNBC cells.

### Thrombin induces p38-dependent pro-inflammatory cytokine expression, while CYLD restrains basal cytokine levels in TNBC cells

**Figure 8:**
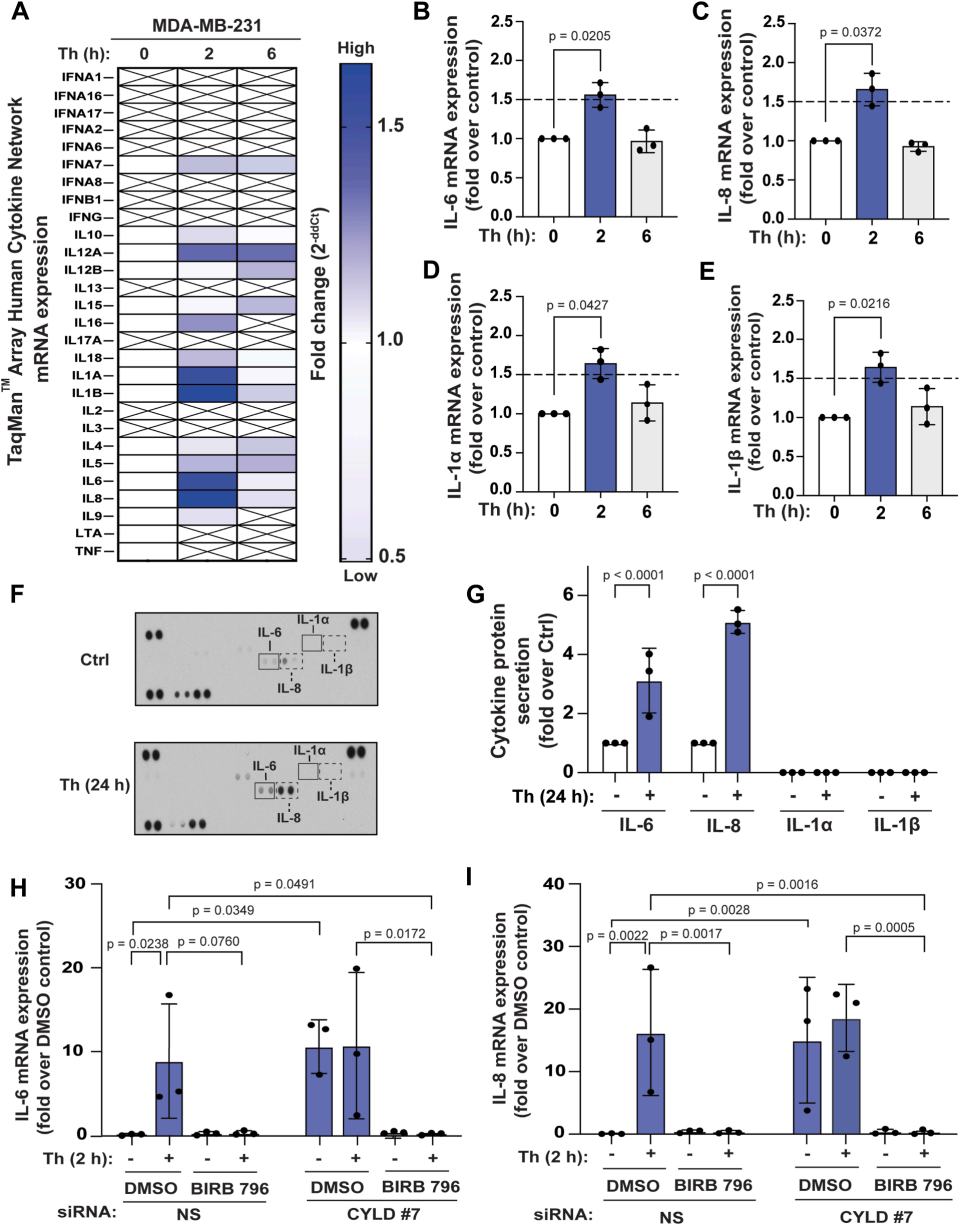
**Thrombin-induced proinflammatory cytokine expression is regulated by p38 and CYLD.***A*, Heat map of 10 nM thrombin (Th)-induced cytokine mRNA transcript expression in MDA-MB-231 cells using the TaqMan Array Human Cytokine Network including 28 different cytokines quantified by reverse transcription quantitative polymerase chain reaction using cycle threshold method. Strikethrough boxes indicate cytokines with very low cycle threshold values ≥ 35 and/or undetectable gene expression. *B–E,* cells were stimulated with Th as indicated and individual gene-specific TaqMan probes were used to verify changes in mRNA expression by reverse transcription quantitative polymerase chain reaction. Changes in mRNA transcript abundance were normalized to 18S rRNA. Data (mean ± S.D., n = 3 independent biological replicates) are expressed as fold over control at 0 min and analyzed by one-way ANOVA. *F,* Th-induced cytokine protein secretion detected using the Proteome Profiler Human Cytokine Array Kit including 36 different cytokines. *G,* changes in cytokine protein secretion were normalized to reference controls according to the manufacturer. Data (mean ± S.D., n = 3 independent biological replicates) are expressed as fold over control and analyzed by one-way ANOVA. *H and I,* cells transfected with NS or CYLD-specific siRNA were pretreated with 50 nM BIRB 796 or DMSO for 1 h prior to stimulation with Th for 2 h. IL-6 and IL-8 mRNA transcript levels were quantified and normalized to 18S rRNA. Data (mean ± S.D., n = 3 independent biological replicates) are expressed as fold change over DMSO control and analyzed by two-way ANOVA. CYLD, cylindromatosis; Th, thrombin; NS, non-specific.

We sought to determine whether the thrombin-induced p38-p65 signaling axis drives an inflammatory transcriptional program and assessed the impact of CYLD. Using a TaqMan Array Human Cytokine Network, we profiled mRNA expression of 28 inflammatory cytokines in MDA-MB-231 cells before and after thrombin stimulation. Of the 28 cytokine genes, IL-6, IL-8, IL-1α, and IL-1β mRNA transcript abundance were the most strongly induced, peaking at 2 h followed by a decline at 6 h after thrombin stimulation (Fig. 8*A*). Individual interleukin gene-specific TaqMan probes were used and confirmed significant induction of the cytokine mRNA expression induced by thrombin at 2 h (Fig. 8, *B*–*E*). We next examined whether these transcriptional changes translated into cytokine protein production. To assess this, we measured secreted cytokines in conditioned media from thrombin-stimulated MDA-MB-231 cells using Proteome Profiler Human Cytokine Array including 36 different cytokines and chemokines. Consistent with the mRNA transcript abundance data, thrombin significantly increased secretion of IL-6 and IL-8, whereas IL-1α and IL-1β remained minimally detectable compared to controls (Fig. 8, *F* and *G*). These results indicate that thrombin preferentially drives production and secretion of a subset of pro-inflammatory cytokines.

Next, we examined whether thrombin-induced cytokine expression depends on p38 and is regulated by CYLD. In control MDA-MB-231 cells, thrombin-induced IL-6 and IL-8 mRNA expression was abolished by the p38 inhibitor BIRB 796 (Fig. 8, *H* and *I*), demonstrating p38 dependence. Given that CYLD negatively regulates basal p38 and p65 phosphorylation, we assessed whether CYLD also modulates cytokine expression. Knockdown of CYLD by siRNA increased basal IL-6 and IL-8 mRNA transcript levels compared to non-specific siRNA control cells (Fig. 8, *H* and *I*), which were similarly suppressed by BIRB 796 compared to control cells (Fig. 8, *H* and *I*). These data suggest that basal cytokine expression in CYLD-deficient cells is p38-dependent. In contrast, thrombin failed to further enhance IL-6 or IL-8 expression in CYLD-deficient cells beyond elevated basal levels, although BIRB 796 suppressed expression under both conditions (Fig. 8, *H* and *I*), indicating near-maximal, p38-dependent cytokine signaling. Together, these results demonstrate that thrombin induces p38-dependent cytokine expression, whereas CYLD restrains basal inflammatory signaling.

### Thrombin promotes p38-dependent MDA-MB-231 cell proliferation, while CYLD restrains basal proliferation and IL-6 contributes to these responses

**Figure 9:**
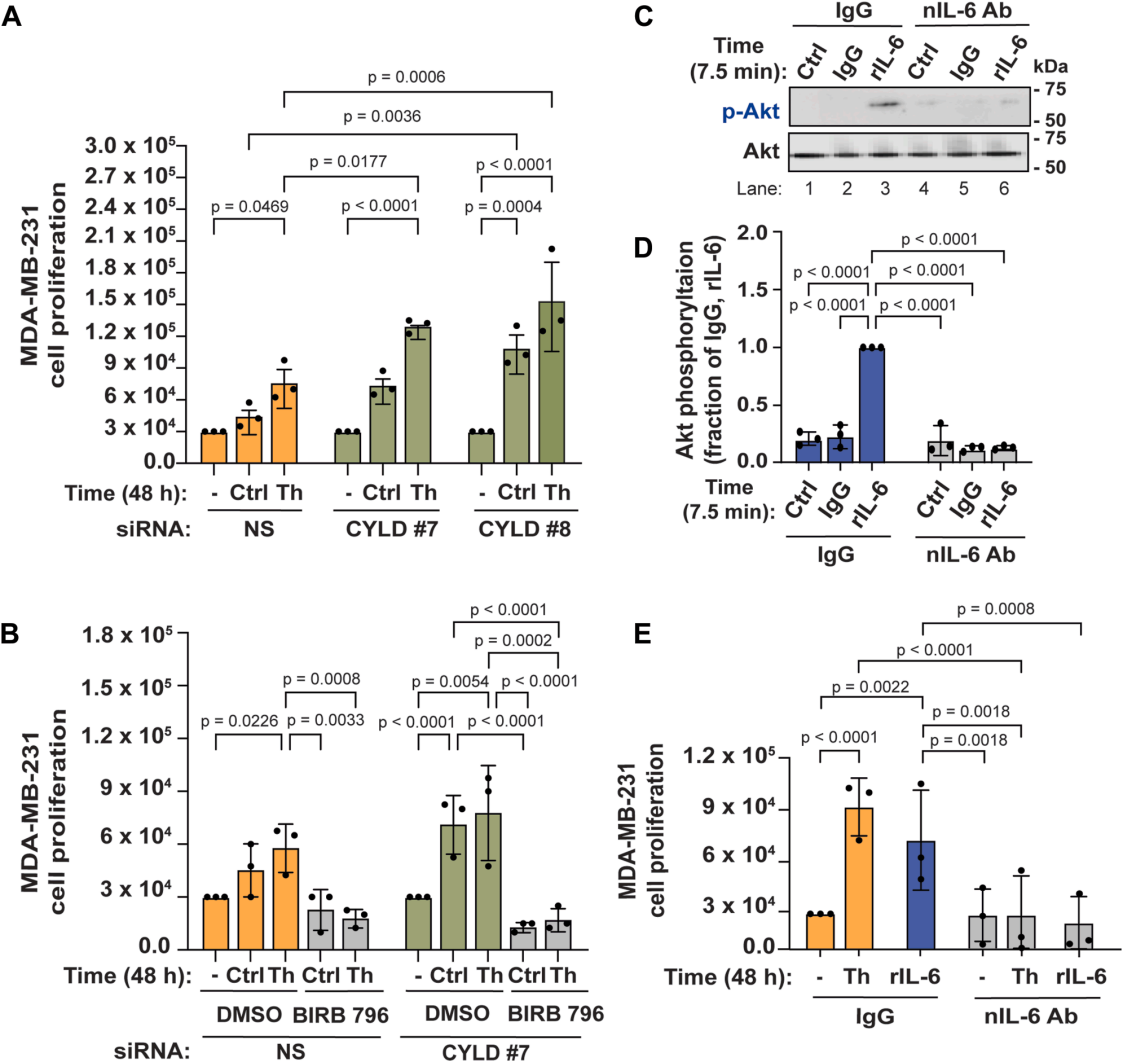
**CYLD regulatesthrombin-induced TNBC cell proliferation, which is mediated by IL-6.***A,* MDA-MB-231 cells transfected with NS or CYLD-specific siRNA were stimulated with 10 nM thrombin (Th) for 48 h, collected and cell proliferation determined. Data (mean ± S.D., n = 3 independent biological replicates) are expressed as an increase in cell number compared to the initial seeding density and analyzed by two-way ANOVA. *B,* cells were transfected with siRNAs as described above, then pretreated with DMSO vehicle or 50 nM BIRB 796 for 1 h prior to Th stimulation for 48 h. Data (mean ± S.D., n = 3 independent biological replicates) are expressed and analyzed as described above. *C and D,* cells were preincubated with 0.5 μg/ml of IL-6 neutralizing antibody (nIL-6 Ab) or IgG control for 1 h, followed by stimulation with 50 ng/ml of recombinant IL-6 (rIL-6) and immunoblotted as indicated. *D,* changes in phospho-Akt was determined after normalization to total Akt protein and expressed as a fraction of rIL-6 preincubated with IgG. *E,* cells were preincubated with IgG control or nIL-6 Ab and then stimulated with Th or rIL-6 as described above at 48 h. Data (mean ± S.D., n = 3 independent biological replicates) were expressed and analyzed as described above. CYLD, cylindromatosis; NS, non-specific; Th, thrombin; TNBC, triple-negative breast cancer.

Thrombin, p38, and p65 promote cell proliferation and CYLD functions as a tumor suppressor (30, 31). However, it remains unknown whether thrombin-induced phosphorylation of p38 and p65 drives cell proliferation and how CYLD regulates it. Depletion of CYLD by siRNA in MDA-MB-231 cells increased basal proliferation to levels comparable to thormbin-treated control cells (Fig. 9*A*), indicating near-maximal proliferation upon CYLD depletion. Despite elevated cell proliferation in CYLD depleted cells, thrombin further promoted cell proliferation (Fig. 9*A*), suggesting that thrombin activates other cell proliferative signaling pathways. We next examined whether p38 activity is required for enhanced basal proliferation and thrombin-induced proliferation observed in CYLD knockdown cells. Control and CYLD-depleted cells were pretreated with or without the p38 inhibitor BIRB 796 or DMSO vehicle control prior to thrombin stimulation. Inhibition of p38 markedly suppressed elevated basal proliferation in CYLD-depleted cells (Fig. 9*B*) as well as thrombin-induced proliferation that were only modestly increased over basal proliferation (Fig. 9*B*). Thus, both basal and modest thrombin-induced proliferation are p38-dependent and restrained by CYLD.

Because thrombin-induced p38-p65 signaling drives inflammatory cytokine expression (Fig. 8), we next examined whether IL-6, a pro-tumorigenic cytokine (8, 32), contributes to proliferation. Since IL-6 stimulates Akt activation (32), we assessed Akt phosphorylation in MDA-MB-231 cells after IL-6 treatment to validate a neutralizing antibody. IL-6 caused a significant increase in Akt Ser473 phosphorylation (Fig. 9*C*, lane 3 *versus* lanes 1–2; Fig. 9*D*), which was blocked by IL-6 neutralization with antibody (Fig. 9*C*, lane 3 *versus* lanes 4–6; Fig. 9*D*). We next evaluated whether IL-6 contributes functionally to thrombin-promoted MDA-MB-231 proliferation. IL-6 increased proliferation to levels similar to thrombin in IgG control treated cells (Fig. 9*E*), whereas IL-6 antibody neutralization attenuated both IL-6 and thrombin-induced proliferation (Fig. 9*E*). Taken together, these findings indicate that thrombin drives MDA-MB-231 proliferation *via* p38 signaling, the loss of CYLD elevates basal proliferation to near-maximal levels, and IL-6 contributes to thrombin-induced proliferation.

### p38-dependent TNBC cell migration is enhanced by thrombin and elevated by CYLD depletion, with contributions from IL-8

**Figure 10:**
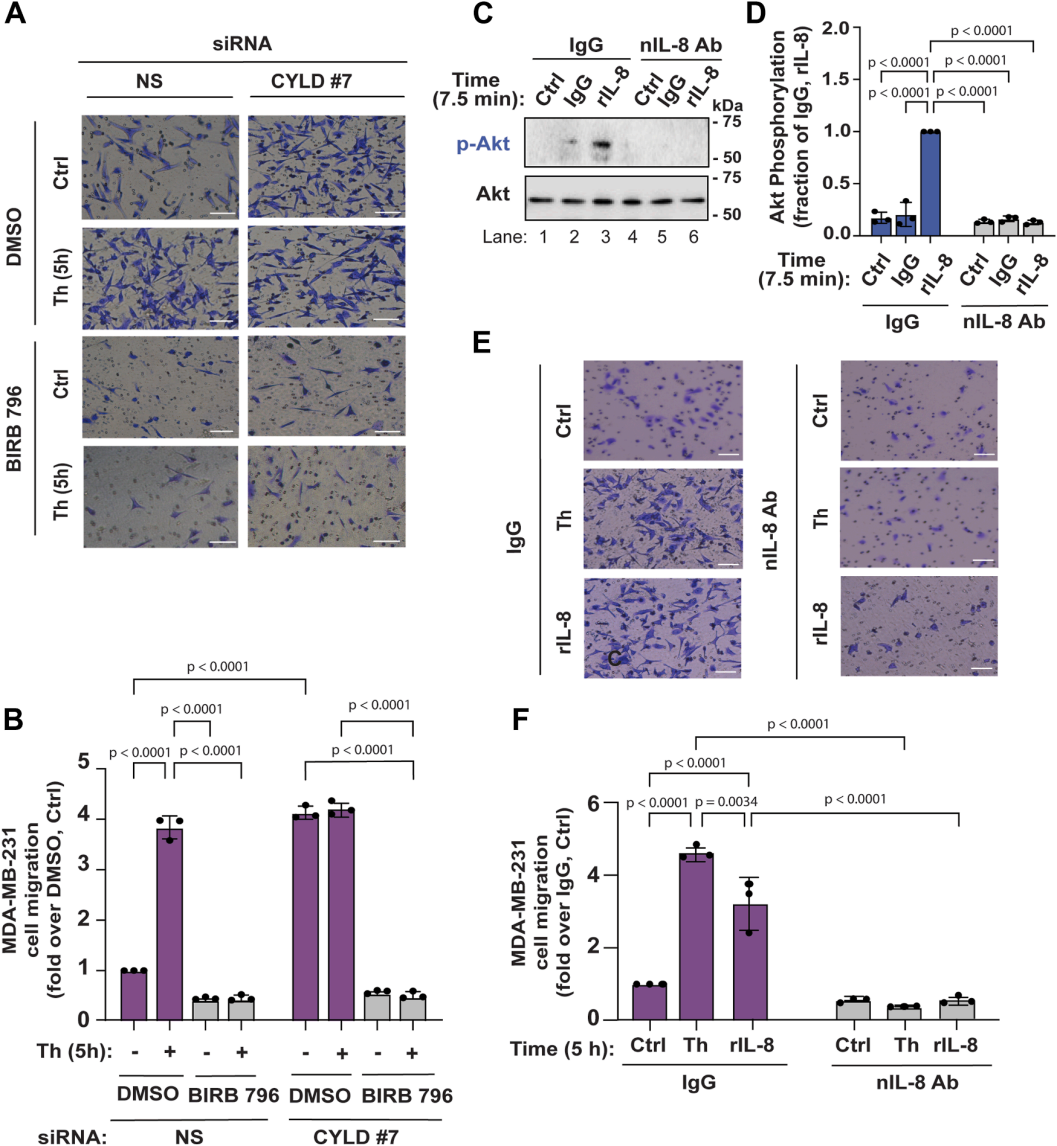
**Thrombin-induced TNBC cell migration is mediated by IL-8 and regulated by CYLD.***A,* MDA-MB-231 cells were transfected with NS and CYLD-specific siRNA and were pretreated with 50 nM BIRB 796 or vehicle control for 1 h and stimulated with 1 pM thrombin (Th). Cell migration was quantified after 5 h of Th treatment. *B,* data (mean ± S.D., n = 3 independent biological replicates) are expressed as fold over DMSO control and analyzed by two-way ANOVA. *C and D,* cells were preincubated with IgG control or 2 μg/ml IL-8 neutralizing antibody (nIL-8 Ab) for 1 h and then stimulated with 50 ng/ml recombinant IL-8 (rIL-8), lysed and immunoblotted as indicated. *D,* changes in phospho-Akt was determined after normalization to total Akt protein and expressed as a fraction of rIL-8 preincubated with IgG. *E,* cells were preincubated with IgG or nIL-8 Ab and then stimulated with Th or IL-8 for 5 h and cell migration was determined. *F,* data (mean ± S.D., n = 3 independent biological replicates) are expressed as fold over IgG control and analyzed by two-way ANOVA. CYLD, cylindromatosis; NS, non-specific; Th, thrombin; TNBC, triple-negative breast cancer.

TNBC exhibits a highly migratory phenotype driven by the phosphorylation of p38 MAPK and NF-κB p65 (11, 33). To assess whether thrombin-induced phosphorylation of p38 and p65 regulates TNBC cell migration and whether this process is controlled by CYLD, MDA-MB-231 cells were transiently transfected with CYLD-specific or non-specific siRNA and treated with thrombin. Thrombin significantly increased cell migration in control cells, while CYLD knockdown elevated basal migration to thrombin-treated levels with no further increase upon stimulation (Fig. 10, *A* and *B*). Because CYLD regulates basal p38 phosphorylation and thrombin increases p38 phosphorylation, we examined whether p38 activity is required for migration. Inhibition of p38 phosphorylation with BIRB 796 markedly reduced cell migration under all conditions, including basal, thrombin-stimulated, and CYLD-depleted cells (Fig. 10, *A* and *B*), indicating a requirement for p38 activity in MDA-MB-231 cell migration.

**Figure 11:**
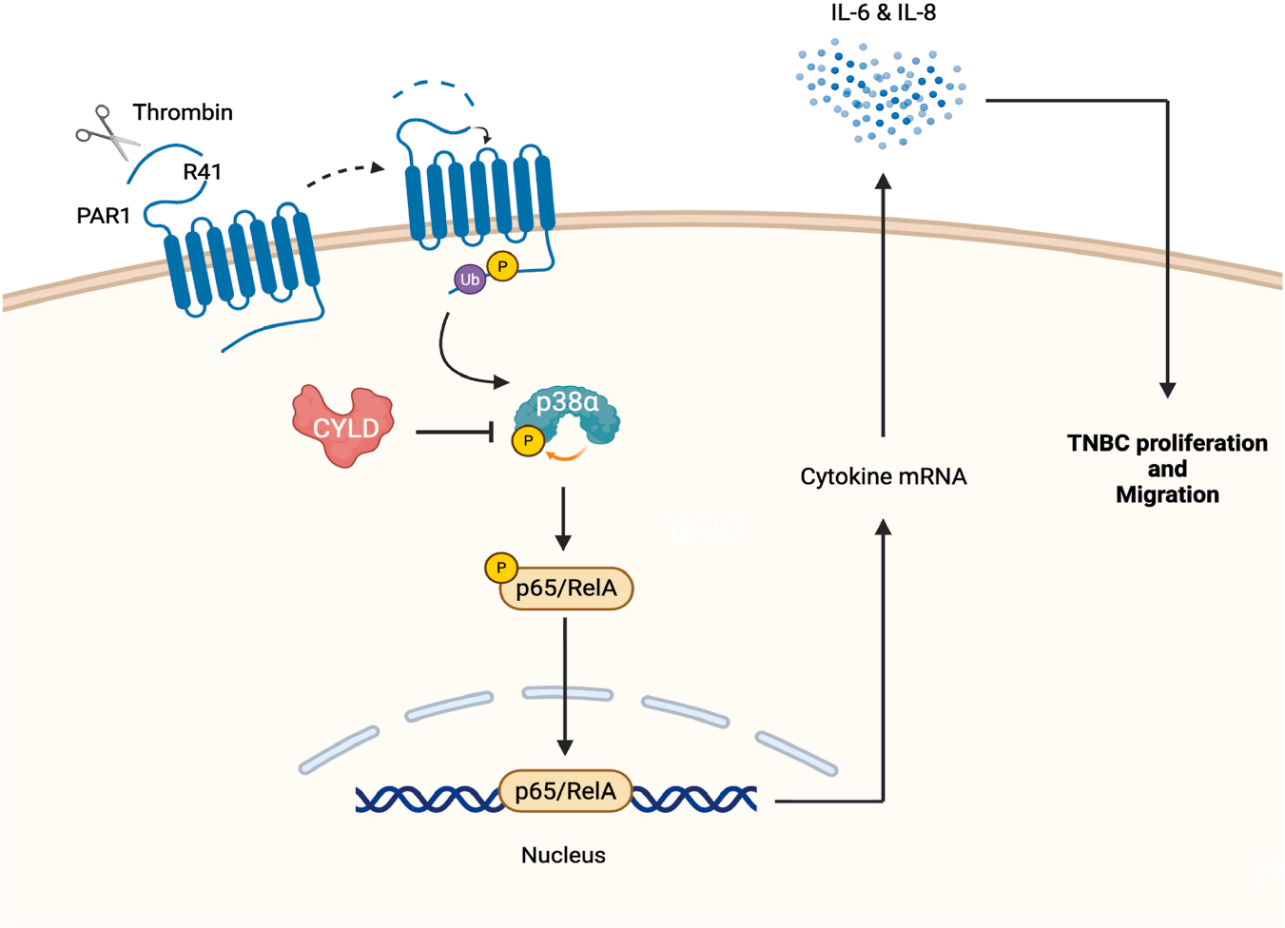
**Model of CYLD regulation ofthrombin-induced p38-p65 signaling axis that drives inflammatory cytokine production and TNBC proliferation and migration.** Th cleaves the N-terminus of PAR1 at R41 and promotes ubiquitin-dependent p38 autophosphorylation and activation of the p65 NF-κB subunit. CYLD functions as a negative regulator of basal p38-p65 activity in TNBC, restraining constitutive pathway activation. Upon thrombin (Th) stimulation, p38-dependent phosphorylation and activation of the p65 subunit result in translocation to the nucleus and drives gene transcription of inflammatory cytokines, including IL-6 and IL-8 secretion, which promotes TNBC cell proliferation and migration, respectively. CYLD, cylindromatosis; TNBC, triple-negative breast cancer.

We next examined whether cytokine production contributes to thrombin-induced migration by examining IL-8, a pro-metastatic cytokine downstream of p38-p65 signaling (8, 34). To evaluate the contribution of IL-8, MDA-MB-231 cells were treated with thrombin or recombinant IL-8 in the presence or absence of an IL-8–neutralizing antibody or IgG control. IL-8 increased Akt phosphorylation (Fig. 10*C*, lane 3 vs lanes 1–2; Fig. 10*D*), which was blocked by preincubation with the IL-8 neutralizing antibody (Fig. 10*C*, lane 3 *versus* lanes 4–6; Fig. 10*D*), indicating that the IL-8 neutralizing antibody is effective. In control cells, thrombin increased migration to levels comparable to recombinant IL-8 stimulation (Fig. 10, *E* and *F*), while the IL-8 neutralization markedly reduced basal, thrombin-, and IL-8-induced migration (Fig. 10, *E* and *F*). Together, these results indicate that CYLD regulates a non-canonical thrombin-activated PAR1 induced p38-p65 signaling axis that promotes inflammatory cytokine production and metastatic phenotypes in TNBC including cell proliferation and migration (Fig. 11).

## Discussion

TNBC is an aggressive breast cancer subtype characterized by high metastatic potential and limited targeted therapeutic options (2, 3). Chronic inflammation contributes to TNBC progression, although the underlying regulatory mechanisms remain unclear. In this study, we define a non-canonical thrombin-PAR1 signaling pathway in TNBC in which p38 MAPK drives NF-κB p65 signaling, inflammatory cytokine production, and cancer progression. Thrombin activates p38 signaling *via* autophosphorylation across multiple TNBC cell lines, which was previously shown to occur through ubiquitin-mediated recruitment of TAB1/TAB2 (13, 20, 24). Thrombin also activates NF-κB p65 signaling downstream of p38 activation, whereas p65 signaling is not required for p38 activation, defining a novel regulatory mechanism for GPCR driven inflammatory signaling.

In canonical NF-κB signaling, p65 activation is coupled to IκBα degradation, enabling nuclear translocation and gene transcription in response to inflammatory stimuli such as tumor necrosis factor cytokines (28). Here we show that thrombin induces robust p65 phosphorylation and nuclear accumulation that is driven by p38 signaling. This process occurs independently of IκBα degradation and involves phosphorylation of both p38 and p65 within a pre-existing p38-p65 complex, supporting p38-mediated regulation of p65 activity. Together, these findings define a non-canonical pathway in which Thrombin-induced p38 activation drives p65 phosphorylation and nuclear translocation, distinct from the classical IκBα-dependent NF-κB pathway.

Thrombin-induced PAR1 ubiquitin-dependent signaling is likely regulated by ubiquitin-modifying enzymes that modulate p38–p65 activity in TNBC cells. While CYLD is known to regulate thrombin-induced p38 MAPK signaling in endothelial cells and HeLa cells (19), here we show in TNBC cells that CYLD restrains basal p38 and p65 phosphorylation, as loss of CYLD elevates both p38 and p65 to levels observed with thrombin. This increase is mediated by p38 signaling and cannot be further enhanced with thrombin stimulation, suggesting a saturated signaling state.

CYLD is a well-established tumor suppressor and negative regulator of NF-κB inflammatory signaling and catalyzes the removal of K63-linked ubiquitin chains from key NF-κB pathway components, including tumor necrosis factor Receptor Associated Factor-2, NF-κB Essential Modulator, Receptor-Interacting Serine/Threonine-Protein Kinase, and Transforming Growth Factor-beta-Activated Kinase-1(22). Through its deubiquitinating activity, CYLD restricts the assembly and activation of ubiquitin-dependent signaling complexes that drive NF-κB activation, thereby preventing IκBα degradation and p65 activation (22). Our findings presented here indicate that CYLD depletion elevates basal NF-κB signaling, whereas thrombin stimulated p65 activation is mediated by upstream p38 signaling. It is not clear if known CYLD NF-κB substrates are involved. In addition, it is unclear how CYLD regulates p38 activity basally and in response to thrombin. Thus, the specific ubiquitinated intermediates and direct CYLD substrates that regulate basal p38 and NF-κB p65 signaling as well as the thrombin activation of the PAR1–p38–p65 signaling axis have yet to be defined.

Cytokine production, a key driver of tumor-associated inflammation and cancer progression, is largely regulated by NF-κB p65 signaling (8, 11, 32). Thrombin similarly promotes pro-inflammatory gene expression (21, 35). In this study, we found in TNBC cells that thrombin induces robust and sustained IL-6 and IL-8 expression, whereas IL-1α and IL-1β are more transient. These effects are mediated by p38-p65 signaling, known regulators of IL-6 and IL-8 expression (21, 34, 36, 37, 38) and restrained by CYLD. Taken together, these findings define a thrombin-driven, p38-p65-mediated inflammatory cytokine program in TNBC that is restrained by CYLD.

Thrombin and CYLD exert opposing effects on tumor-associated phenotypes such as proliferation and migration. Th acts as a pro-tumorigenic factor that promotes proliferation, an effect amplified by CYLD loss, consistent with CYLD’s tumor-suppressive function. CYLD depletion elevated basal proliferation through p38-dependent signaling to levels approaching thrombin stimulation, consistent with the observed increase in basal p38 autophosphorylation and downstream p65 phosphorylation. While p38 signaling remained a major contributor to the proliferative phenotype, as its inhibition markedly reduced proliferation, in some cases an additional increase was observed following thrombin stimulation in CYLD-depleted cells suggesting that other thrombin-activated PAR1 signaling pathways also engage additional pro-survival pathways. Previous studies have shown that thrombin/PAR1 activates ERK1/2 (39) PI3K-Akt (40), and ROCK-RHOA (41) signaling pathways, which may further enhance proliferation beyond p38 signaling. Because thrombin-induced p38-p65 signaling promotes both inflammatory cytokine expression and proliferation, cytokines downstream of this pathway may also likely contribute to the proliferative response. Among the cytokines induced by thrombin, IL-6 is most directly linked to proliferation (8, 10). Consistent with this, IL-6 promotes Ser473 Akt phosphorylation and proliferation, while its neutralization with antibody attenuates both IL-6 and thrombin-induced proliferation, indicating that IL-6 is a key downstream mediator of thrombin-driven proliferative signaling.

Cell migration is also induced by thrombin stimulation (17), although CYLD’s role in this context has not been clearly defined. Our findings indicate that CYLD acts as a suppressor of thrombin-induced migration in TNBC cells, as its loss enhances p38-driven inflammatory signaling and migratory behavior in a p38-dependent manner. While distinct cytokines differentially link inflammation to tumor phenotypes, migration is primarily associated with IL-8 signaling (8, 42). Antibody neutralization of IL-8 reduces TNBC migration, whereas thrombin or recombinant IL-8 enhances migration and Akt phosphorylation, which is reduced by IL-8 blockade. Together, these findings support a model in which thrombin-induced p38 activation drives TNBC proliferation and migration through cytokine signaling, with CYLD acting as a negative regulator of the p38-p65 NF-κB inflammatory signaling axis (Fig. 11).

## Experimental procedures

### Antibodies and reagents

Antibodies were obtained from commercial sources, and detailed information is provided in Table S1. Human α-Th, pharmacological inhibitors, cytokines and other reagents are listed in Table S2.

### Cell culture

MDA-MB-231, BT549, and Hs578T cells were obtained from ATCC and cultured under standard conditions. Cells were routinely tested for *mycoplasm*a contamination using Venor GeM *Mycoplasma* detection kit per the manufacturer’s instructions. Cell culture medium and culturing conditions are provided in Tables S4 and S5.

### siRNA transfection

Transfections with siRNAs were performed as previously described (21). Briefly, MDA-MB-231 or BT549 cells (1 × 10^5^ cells/well) were seeded in 12-well plates and grown overnight. The following day, cells were transfected with 20 nM target-specific or non-specific siRNAs using Oligofectamine 2000 according to the manufacturer’s instructions. After 48 h, cells were either left untreated or pretreated with 50 nM BIRB 796 and stimulated with or without 10 nM thrombin for the indicated time points. Cells were then harvested for protein extraction and analyzed by Western blot. Sequences of individual siRNAs are listed in Table S3.

### Signaling assays

Signaling assays were performed as previously described (21). Briefly, cells were serum-starved overnight in their respective media containing 10 mM Hepes, 1 mM CaCl_2_, and 1 mg/ml bovine serum albumin (BSA). Following serum starvation, cells were incubated with either 10 μM Vorapaxar, 50 nM BIRB 796, 2.5 μM BAY 11-7082, 30 μM Fasudil, or 0.1% DMSO (vehicle control) for 1 h at 37 °C. Cells were then stimulated with 10 nM thrombin for the indicated times. For cytokine signaling experiments, cells were incubated with an IL-6 neutralizing antibody (0.5 μg/ml) or an IL-8 neutralizing antibody (2 μg/ml) for 1 h, followed by stimulation with recombinant IL-6 or IL-8 (50 ng/ml) for 7.5 min at 37 °C. After stimulation, cells were lysed and samples were processed and immunoblotted as described below.

### Western blot analysis

Western blotting was performed as previously described (21). Briefly, cells were lysed in 2X Laemmli sample buffer (125 mM Tris-HCl, pH 6.5, 5% SDS, 20% glycerol, 0.003% bromophenol blue) containing 200 mM DTT with protease inhibitors including 1 μg/ml of leupeptin, aprotinin, trypsin protease inhibitor and pepstatin, and 100 μg/ml benzamidine and phenylmethylsulfonyl fluoride. Cell lysates were denatured at 95 °C for 5 min, resolved on a 9% SDS-polyacrylamide gel and transferred to 0.45 μm PVDF membranes. Membranes were blocked with 5% BSA in 1X Tris-buffered saline with Tween-20 (TBST; 50 mM Tris-HCl [pH 7.4], 150 mM NaCl, and 0.1% [v/v] Tween-20) and incubated overnight at 4 °C with primary antibodies. After washing, membranes were incubated with HRP-conjugated secondary antibodies for 1 h at room temperature. Signals were detected using chemiluminescence and a ChemiDoc MP Imaging System (Bio-Rad). The protein band intensities were quantified by ImageJ and normalized to their respective total protein.

### Immunofluorescence staining

MDA-MB-231 cells (1 × 10^5^ cells/well) were seeded onto fibronectin-coated glass coverslips in 12-well plates and serum-starved for 24 h. Cells were incubated with fasudil (30 μM) for 1 h, followed by pretreatment with either BIRB 796 (50 nM) or BAY 11-7082 (2.5 μM) for an additional 1 h at 37 °C. Cells were then stimulated with or without thrombin (10 nM) for 7.5 min at 37 °C. Following treatment, cells were washed with 1X PBS and fixed with 4% paraformaldehyde for 10 min at room temperature. Cells were washed again with 1X PBS and permeabilized with 0.5% Triton X-100 in 1X PBS for 10 min at room temperature. After permeabilization, cells were washed and blocked with 5% BSA in PBS for 1 h. The anti-p65 antibody (1:400) was applied and incubated overnight at 4 °C. Secondary antibody goat anti-rabbit IgG conjugated to Alexa Fluor 488 (1:400) was incubated for 1 h at room temperature. Nuclei were visualized with DAPI staining. Coverslips were mounted with ProLong Gold Antifade Mountant. Confocal image acquisition was performed using an Olympus IX81 DSU spinning-disk microscope equipped with a 60x Plan Apo objective lens (numerical aperture = 1.4), along with appropriate excitation–emission filters and a Cool SNAP HQ2 CCD camera (Andor) using Metamorph, version 7.7.4.0 software (Molecular Devices; http://www.moleculardevices.com/Products/Software/Meta-Imaging-Series/MetaMorph.html). Images were processed using Fiji plugin (ImageJ) and the number of cells with p65 nuclear staining were quantified from three independent experiments using six images per condition.

### Co-IP

MDA-MB-231 cells (1 × 10^5^ cells/well) were seeded and serum-starved for 24 h. After starvation, cells were treated with 10 nM thrombin for the indicated time points at 37 °C. Cells were then lysed in Triton lysis buffer (50 mM Tris [pH 7.4], 100 mM NaCl, 5 mM EDTA, 1% v/v Triton X-100, 50 mM NaF, and 10 mM NaPP) supplemented with protease inhibitors. Protein A/G Plus-Agarose beads were pre-blocked in 5% BSA for 30 min at 4 °C and washed prior to use. Cell lysates were precleared with pre-blocked Protein A/G Plus-Agarose beads for 1 h at 4 °C. Precleared lysates were incubated overnight at 4 °C with anti-p38 or anti-p65 antibodies. The following day, pre-blocked Protein A/G Plus-Agarose beads were added for 1 h at 4 °C to capture immune complexes. Immunoprecipitates were washed with 1X TBST, eluted in 2X LSB containing 200 mM DTT, resolved by SDS-PAGE, and transferred to PVDF membranes. Membranes were immunoblotted with anti-phospho-p38 Thr180/Tyr182 or anti-phospho-p65 Ser536 antibodies, as indicated. Immunoprecipitated phosphoproteins were quantified and normalized to total p38 or total p65 as described above.

### Proliferation assays

MDA-MB-231 cells (3 × 10^4^ cells per well) were seeded in 12-well plates and grown for 24 h. Cells were then transfected with either 20 nM CYLD siRNA or non-specific siRNA using Oligofectamine 2000 for 48 h. Following transfection, cells were serum-starved for 24 h and pretreated for 1 h with 50 nM BIRB 796, recombinant IL-6 or IL-8 (50 ng/ml), IL-6 neutralizing antibody (0.5 μg/ml) or IL-8 neutralizing antibody (2 μg/ml), or 0.1% DMSO (vehicle control) at 37 °C, as indicated. Cells were then stimulated with 10 nM thrombin and incubated for an additional 48 h in the continued presence of Th and/or the indicated inhibitor, recombinant cytokine, or neutralizing antibody at 37 °C. After treatments, cells were collected and counted to assess cell proliferation compared to the initial seeding density.

### Reverse transcription quantitative polymerase chain reaction

RNA was isolated from MDA-MB-231 cells seeded at 1 × 10^5^ cells per well in a 6-well plate grown overnight using Direct-zol RNA Miniprep Plus Kit. Total RNA was quantified, and 1 μg of total RNA was used for cDNA synthesis reaction with the SuperScript IV VILO Master Mix with ezDNase enzyme kit. Reverse transcription quantitative polymerase chain reaction was performed with TaqMan™ Fast Advanced Master Mix on a QuantStudio 3 Real-Time PCR System (Thermo Fisher Scientific). TaqMan gene-specific probes are listed in Table S2. Cytokine mRNA transcript levels were analyzed using the comparative cycle threshold (CT) (threshold cycle) method, normalized to 18S ribosomal RNA expression. Briefly, the number of Ct was determined for each target, and the Ct value for 18S was subtracted from the Ct value for each target, the differences in expression was then determined using the 2-ΔΔCt method.

### Human cytokine arrays

TaqMan Array Human Cytokine Network (Applied Biosystems) containing 28 cytokines was used to screen Th-induced cytokine mRNA transcript expression in MDA-MB-231 cells. Briefly, cells were seeded in a 12-well plate (8 × 10^4^ cells/well) and grown overnight. Cells were then serum-starved as described above and treated with 10 nM thrombin for the indicated time points. RNA was extracted and purified, cDNA synthesized, and reverse transcription quantitative polymerase chain reaction was performed and quantified as described above.

Proteome Profiler Human Cytokine Array Kit (R&D Systems) including 36 cytokines and chemokines was used to detect thrombin-induced secreted cytokines. Briefly, MDA-MB-231 cells were seeded in 10 cm dishes at 7.5 × 10^5^ cells per dish and grown overnight. Cells were then serum-starved overnight, treated with or without Th in serum-free medium for 24 h. Supernatant was collected, centrifuged and used as conditioned media. Conditioned media was incubated with a cocktail of biotinylated detection antibodies and then applied to nitrocellulose membranes pre-spotted with 36 biotinylated cytokine and chemokine capture antibodies in duplicate and incubated overnight at 4 °C. After overnight incubation, membranes were washed and incubated with streptavidin–HRP and developed by chemiluminescence. Signals were visualized using ChemiDoc MP Imaging System and quantified using ImageJ (https://imagej.net/ij/). Cytokine positive signals were determined by subtracting background from an unspotted area, averaging the duplicate positive signals and normalized to reference spots across independent experiments.

### Transwell migration assay

MDA-MB-231 cell migration was assessed using Transwell chambers (8-μm pore size) coated with 1 μg/ml of collagen. Serum-starved cells were detached using Corning CellStripper Dissociation Reagent, resuspended in serum-free medium, and seeded into the upper chamber in the presence of the indicated treatments. The lower chamber contained medium with 5% BSA. After incubation for 5 h at 37 °C, nonmigrating cells were removed from the upper surface, and the migrated cells on the lower membrane surface were fixed with 4% paraformaldehyde and stained with 0.2% crystal violet dye in methanol. Membranes were washed, dried, and imaged under a microscope. The number of migrating cells was quantified from images taken from six random fields per condition from treated and untreated control cells using Fiji plugin (ImageJ).

### Software

Schematics and Figures were created in Adobe Illustrator and Photoshop. The cartoons were created with BioRender.com. ImageJ was used to quantify western blots and Fiji plugin (ImageJ) was used for image processing and quantification.

### Statistical analyses

All statistical analyses were performed in Prism 10.2 statistical software (GraphPad Software; https://www.graphpad.com/guides/prism/9/user-guide/index.htm). Statistical test analysis methods and *p* values are indicated in the figure legends.

## Data availability

The data supporting the findings of this study are available from the corresponding author upon reasonable request.

## Supporting information

This article contains supporting information.

## Conflict of interest

The authors declare that they have no conflicts of interest with the contents of this article.
