# Supplementary material for: The deubiquitinase CYLD inhibits thrombin-induced p38 MAPK–p65 NF-κB signaling and inflammatory cytokine production to suppress triple-negative breast cancer progression
Source: J Biol Chem. 2026 Jul 16;302(9):113341. doi: 10.1016/j.jbc.2026.113341 (PMC13487619; doi:10.1016/j.jbc.2026.113341)
Supplement: Supplementary file 1 [file mmc1.docx]

**Supporting Information**

**Supplementary Table S1. Antibodies**

| **Target** | **Application** | **Company** | **Catalog #** | **Clone** |
| --- | --- | --- | --- | --- |
| IκBα | WB | Cell Signaling Technology | #4814 | L35A5 |
| p38 MAPK | WB | Cell Signaling Technology | #9212 | — |
| Phospho-p38 (Thr180/Tyr182) | WB | Cell Signaling Technology | #4511 | D3F9 |
| p65 (NF-κB) | WB/IF | Cell Signaling Technology | #8242 | D14E12 |
| Phospho-p65 (Ser536) | WB | Cell Signaling Technology | #3033 | 93H1 |
| Akt | WB | Cell Signaling Technology | #9272 | — |
| Phospho-Akt (Ser473) | WB | Cell Signaling Technology | #4060 | D9E |
| CYLD | WB | Cell Signaling Technology | #8462 | — |
| Goat anti-Rabbit IgG-HRP | WB | Bio-Rad | #1706515 | — |
| Goat anti-Mouse IgG-HRP | WB | Bio-Rad | #1706516 | — |
| Anti-β-Tubulin Mouse | WB | Cell Signaling Technology | #86298 | — |
| Alexa Fluor 488 anti-Rabbit | WB | Invitrogen | #A-11034 | — |
| IL-8 antibody | Neutralizing Antibody | R&D Systems | #MAB208 |  |
| IL-6 antibody | Neutralizing Antibody | Thermo Fisher Scientific | #MA5-23698 |  |

Abbreviations: Western blot (WB), immunofluorescence (IF)

**Supplementary Table S2. Reagents, Inhibitors, Cytokines, and Kits**

| **Reagent** | **Function** | **Company** | **Catalog #** |  |
| --- | --- | --- | --- | --- |
| Human α-Thrombin | PAR1 agonist | Enzyme Research Laboratories | #HT1002a |  |
| Vorapaxar | PAR1 antagonist | Axon Medchem | #1755 |  |
| BIRB 796 | p38 inhibitor | Sigma-Aldrich | #506172 |  |
| BAY 11-7082 | NF-κB inhibitor | MedChemExpress | #HY-13453 |  |
| Fasudil | ROCK inhibitor | LC Laboratories | #H-2330 |  |
| IL-6 recombinant protein | Cytokine | PeproTech | #200-06-50UG |  |
| IL-8 recombinant protein | Cytokine | PeproTech | #200-08-50UG |  |
| DAPI | IF | Thermo Fisher Scientific | #D-1306 |  |
| Direct-zol RNA Miniprep Plus Kit | RNA isolation | Zymo Research | #R2072 |  |
| SuperScript IV VILO Master Mix | cDNA synthesis | Thermo Fisher Scientific | #111766050 |  |
| TaqMan Fast Advanced Master Mix | qPCR | Thermo Fisher Scientific | #4444964 |  |
| TaqMan^TM^ Array Human Cytokine Network | qPCR | Applied Biosystems | #4414124 |  |
| Human Cytokine Array Kit | Cytokine profiling | R&D Systems | #ARY005B |  |
| Oligofectamine 2000 | siRNA transfection reagent | Thermon Fisher Scientific | #12252011 |  |
| TaqMan IL-6 probe | mRNA transcript expression | Thermo Fisher Scientific | #Hs00174131_m1 |  |
| TaqMan IL-8 probe | mRNA transcript expression | Thermo Fisher Scientific | #Hs00611330_m |  |
| TaqMan IL-1α probe | mRNA transcript expression | Thermo Fisher Scientific | #Hs00215725_m1 |  |
| TaqMan IL-1β probe | mRNA transcript expression | Thermo Fisher Scientific | #Hs01651394_s1 |  |
| TaqMan 18S rRNA probe | RNA transcript expression | Thermo Fisher Scientific | #Hs03003631_g1 |  |
| Venor GM Mycoplasma Detection Kit | Mycoplasma contamination | Sigma-Aldrich | #MP0025-1KT |  |
| Prolong Gold Antifade Mountant | Slide Mountant | Invitrogen | #P36934 |  |
| Transwell Chambers | Cell migration | Corning | #353097 |  |
| Cell Stripper | Cell dissociation reagent | Corning | #MT25056CI |  |
| Crystal Violet Dye | Cell stain | Sigma-Aldrich | #C3886-256 |  |
| Protein A/G Plus-Agarose beads | Co-IP | Santa Cruz Biotechnology | #sc-2003 |  |

**Supplementary Table S3. siRNA Sequences**

| **Target** | **siRNA Name** | **Sequence (5’→3’)** | **Company** | **Catalog #** |
| --- | --- | --- | --- | --- |
| CYLD | CYLD #7 | GGAGAGUACUUGAAGAUGU | Dharmacon | #J-004609-07 |
| CYLD | CYLD #8 | GAAGGUUGGAGAAACAAUA | Dharmacon | #J-004609-08 |
| p65 (RELA) | p65 #5 | AAGATCAATGGCTACACAGGA | Qiagen | SI00301672 |
| p65 (RELA) | p65 #8 | ATGGAGTACCCTGAGGCTATA | Qiagen | SI02663101 |
| p38 (MAPK14) | p38 #5 | AACTGCGGTTACTTAAACATA | Qiagen | S100300769 |
| p38 (MAPK14) | p38 #6 | CAGAGAACTGCGGTTACTTAA | Qiagen | S100605157 |
| Control | AllStars siRNA | — | Qiagen | #1027281 |

**Supplementary Table S4. Cell Lines and Culture Conditions**

| **Cell Line** | **Source** | **Medium** | **Supplements** | **Supplement Company (Catalog #)** | **Culture Conditions** |
| --- | --- | --- | --- | --- | --- |
| MDA-MB-231 | ATCC | Leibovitz’s L-15 | 10% FBS | Gibco (#10437-028) | 37°C, no CO₂ |
| BT549 | ATCC | RPMI | 10% FBS | Gibco (#10437-028) | 37°C, 5% CO₂ |
| Hs578T | ATCC | DMEM | 10% FBS | Gibco (#10437-028) | 37°C, 5% CO₂ |

**Supplementary Table S5. Culture Medium**

| **Cell Line** | **Medium** | **Medium Company (Catalog #)** |
| --- | --- | --- |
| MDA-MB-231 | Leibovitz’s L-15 | Thermo Fisher Scientific (#11415-064) |
| BT549 | RPMI | Thermo Fisher Scientific (#11875-093) |
| Hs578T | DMEM | Corning (#10-013-CV) |
